# Health insurance coverage and access to care in China

**DOI:** 10.1186/s12913-022-07498-1

**Published:** 2022-02-03

**Authors:** De-Chih Lee, Jing Wang, Leiyu Shi, Caroline Wu, Gang Sun

**Affiliations:** 1 Department of Information Management, Da-Yeh University, No.168, University Rd., Dacun, Changhua 515006 Taiwan, R.O.C.; 2 Johns Hopkins Primary Care Policy Center, Baltimore, MD 21205 USA; 3 Anhui Medical University, Hefei, 230032 China; 4 Johns Hopkins Bloomberg School of Public Health, Baltimore, MD 515006 USA; 5 Department of Health Management, School of Health Management, Southern Medical University, Guangzhou, Guangdong 510515 P.R. China

## Abstract

**Objective:**

The study examined the relationship between health insurance coverage and access to needed healthcare including preventive, primary, and tertiary care among Chinese adult population.

**Data and methods:**

Data for this study came from the 2018 China Health and Retirement Longitudinal Study (CHARLS), a population-based probability sample survey. Key measures included insurance coverage (high-, moderate-, low- and no-insurance), access to care (physical examination, physician visit, office visit, inpatient care, and satisfaction with care), and personal sociodemographics. Multiple-factor generalized linear mixed model was applied to estimate the odds ratio (OR) and the 95% confidence interval (CI) of HI coverage for the four indicators of access to care, after controlling for individual characteristics and aggregation among different villages.

**Results:**

The majority of Chinese adults had some health insurance with only 3.15% uninsured. However, most had low-coverage insurance (64.82%), followed by moderate-coverage insurance (16.70%), and high-coverage insurance (15.33%). Health insurance was significantly and positively associated with access to needed healthcare (preventive, primary, and tertiary). There was also a significant gradient association between extent of insurance coverage and access to care.

**Conclusion:**

Not only health insurance mattered in enhancing access to care but that there was a significant gradient association between extent of insurance coverage and access to care with higher coverage relating to better access.

## Introduction

Health insurance (HI) is part of social security that assures the provision of necessary health services to citizens in return for periodic pecuniary tax contributions [1]. Managed care and strategic purchasing options have been promoted as potential approaches to furthering the equity, access, and quality of recipient healthcare [2]. Both economics theory [3] and a large body of literature support the relationship between insurance coverage and access to care [4–6] as well as satisfaction with the care experience [7–9]. Five major areas are considered in evaluating the performance of HI policies: quality, accessibility, efficiency, continuity, and fairness [10]. Both private and public sectors provide options for HI coverage; a well-known federal welfare subsidiary example is Medicare and Medicaid within the United States (US).

Inadequate or decrepit HI for the general population translates to a limitation in access to healthcare for individuals who may benefit most [11]. Any gain or buttressing of both access to care and insurance coverage could also improve citizens’ quality of life. Improved insurance coverage would better support the attainment of three goals: development of reliable sources of care for each individual, expansion of access to medical, dental and vision care, and improvement of patient satisfaction within each care area [12]. Previous studies have also found a positive relationship between expansion in HI and increase in patient satisfaction and quality of care [13–19].

The United States health system illustrates the inadequate access to healthcare. A recent study of a population of 100,667 individuals within the US showed that foreign-born heterosexual adults within the country were associated with higher rates of absent or inadequate medical insurance coverage [14]. Another study for adults aged 18–64 using data from the 2016–2018 Medical Expenditure Panel Survey (MEPS) found that adults with limited English proficiency showed more significant increases in the attainment of a consistent source of care, than did adults with high English proficiency (5 percentage points, *p* = 0.007). Adults with limited English proficiency also saw more significant increases in rates of obtaining medical and dental care than their high proficiency counterparts (1.4 percentage points, *p* = 0.013, and 2.8 percentage points, *p* = 0.009, respectively) [15]. Compared with community health center (CHC) patients without insurance, CHC patients with insurance coverage were more likely to have access to necessary medical care (OR = 2.12) [16]. Medicaid expansion within the US was also associated with significant reductions in time-to-care (− 16 percentage points, *p* < 0.01) and significant reductions in number of individuals delaying attainment of medications due to cost (− 18 percentage points, 95% confidence interval: − 27 to − 9, *p* < 0.001) [17]. Increased health insurance coverage also contributed to a reduction in the probability of patients perceiving an unmet need for mental health treatment by 2.2 percentage points (*p* < 0.05) [18]. In P.R. China, an investigation found that more than 80% of returning migrant workers had medical insurance most of which was the new rural cooperative medical insurance [19]. Participating in medical insurance had significant effects on their medical seeking behavior.

Despite recent improvements in coverage, disparities still exist. Immigrants from Mexico to the US had high uninsured rates and inadequate access to care [20]. In fact, 62% of surveyed Mexican individuals living within the US in 2013 had no regular source of care and instead relied on private clinics and pharmacies [20]. Nonelderly adults with HI coverage in Massachusetts often reported additional financial barriers to care and difficulties paying for medical bills compared to the elderly [21]. Some data showed no improvements in time-to-care, access to mental or behavioral health services, or establishment of a consistent source of care for patients in US following the passage and subsequent expansion of HI under the Patient Protection and Affordable Care Act in 2012–2015 [22]. The information and data of Zhejiang and Gansu provinces in P.R. China in 2008 reflected that the new rural cooperative medical system had not effectively improved the use of relevant medical services, and most of the policyholders of the new rural cooperative medical system were resistant to the hospital outpatient service [23].

Regarding correlation between insurance coverage and patient satisfaction on an international level, a study from Ghana indicated that a higher proportion of insured patients reported “very satisfied” with waiting times at clinics compared to their uninsured patient counterparts (21% vs. 19%), and reported “very satisfied” with waiting times at laboratory and x-ray departments compared to the same uninsured patient counterparts (27 vs. 22%) [24]. There was no reported significant difference in patient satisfaction with quality of care received between the insured and uninsured groups (OR = 0.43, *P* > 0.05) [24].

In China, health outcomes and coverage percentage are influenced by location. In rural areas, HI is less expensive but comprises a higher percentage of a family’s average income. A 2017 study evaluating 1783 individuals in Jaingsu Province noted that urban area residents spent three-fold per capita on healthcare when compared to their rural counterparts. Rural area residents reported worse health service performance in vaccination, maternal and prenatal care, tuberculosis testing and treatment, smoking cessation, and antihypertensive treatments [25]. Despite this poor performance, rural individuals reported more than 30% of household income on catastrophic healthcare. The Chinese National Health Survey (CNHS) reported in 2003 that 13% of urban residents and 19% of rural residents did not seek outpatient care due to cost. Another 2018 study evaluated, through time-series regression analysis, the quantity and quality of medical services supplied before and after the implementation of China’s universal medical insurance system (UMIS) [26]. By 2014, UMIS had covered 97.5% of the entire population and hospital inpatient and outpatient visits were increased. The average fatality rate of inpatients decreased significantly across all measured hospitals. However, despite this increased access to care and uptick in quality of service, low-income patients remained at-risk for catastrophic payments after hospital stays. Affordability under UMIS is still relatively weak, especially in emergent care [26].

In this article, we aim to expand upon the direct relationship between HI coverage and access to needed healthcare in P.R. China, based on 2018 data from the China Health and Retirement Longitudinal Study (CHARLS), the latest year the dataset was made publicly accessible at the time of study. The CHARLS outlines variations and inconsistencies in access to care among insured and uninsured adults. Findings will enrich the literature on extent of HI coverage and access to needed healthcare, inform the development of future health policy, and guide national healthcare reform efforts.

## Methods

### Data

Data for this study came from the CHARLS project, a large-scale interdisciplinary survey project sponsored by the National Development Research Institute of Peking University and jointly implemented by China Social Science Survey Center of Peking University and Youth League Committee of Peking University in 2018 (http://charls.pku.edu.cn/pages/about/charls/zh-cn.html). Probabilities proportional to size were applied in the sampling of 150 counties within 30 Chinese provinces, excluding the Tibet Autonomous Region, Taiwan province, and Hong Kong and Macao special administrative regions. Three villages were then randomly selected from each county totaling 450 villages. Eighty households were randomly selected from each village and investigated; the number of households required to power the study was determined by pre-survey participation and refusal rates (11.17%) for 24 households sampled within each village. A total of 19,816 individuals were selected for the final sample. A filter questionnaire selected individuals age 45 and over and excluded those less than age 45.

### Measures

#### Health insurance coverage

Interviewees were asked if they were the policy holder or primary beneficiary to any type of HI. In order to analyze responses that reflect type of insurance coverage, interviewee insurance status was recoded into four categories based on extent of coverage: (1) high-coverage insurance scheme (including urban employee medical insurance (yi-bao) only, government medical insurance only, private medical insurance purchased by work unit only, or private medical insurance purchased by individual only) which covered about 80% of the cost of medical care; (2) moderate-coverage insurance scheme (including urban and rural resident medical insurance only or urban resident medical insurance only) which covered 50–80% of the cost of medical care; (3) low-coverage insurance scheme (including the new rural cooperative medical insurance (he-zuo-yi-liao) only) which covered less than 50% of the cost of medical care, and (4) No insurance. The related items in the CHARLS questionnaire (http://charls.pku.edu.cn) used to construct this measure are: 1. “How much did you spend for all the doctor visits during last month? (Include both out-of-pocket payment and insurance payment paid part and insurance reimbursement.)” 2. “How much did you spend out-of-pocket for all the doctor visits during last month?” We then calculated the percentage of the cost covered by the insurance scheme based on the responses to these two items.

#### Access to care

Four indicators of access to care were used in this study drawn from the data representing accessing to preventive care, primary care, tertiary care, and satisfaction with the care experience: (1) completion of at least one physical examination with responses of “had” or “had never” (valid frequencies were 9253 or 47.56% and 10,203 or 52.44%, respectively); (2) attendance of a medical office visit during the last month with responses of “yes” or “no” (valid frequencies were 3204 and 16,612, respectively); (3) receipt of inpatient care in the past year with responses of “yes” or “no” (valid frequencies were 3327 and 16,437, respectively); and (4) satisfaction with the quality, cost, and convenience of local medical services with responses of “satisfied” or “dissatisfied” (valid frequencies were 15,904 and 3095, respectively). In this study, regular physical examination was treated as a preventive care measure. Although government policy recommends its use, its actual implementation could still vary significantly by individual and insurance characteristics. The variable was also used due to lack of more appropriate measures of preventive care, a limitation of the dataset itself.

#### Individual characteristics

Some important individual characteristics potentially associated to insurance coverage and/or healthcare experience were included: (1) age-group with responses of “< 64” or “≥65” (valid frequencies were 12,061 or 61.73% and 7478 or 38.27%, respectively); (2) gender with responses of “male” or “female” (valid frequencies were 9340 or 47.13% and 10,476 or 52.87%, respectively); (3) residence with responses of “the center of city/town” (urban), “combination zone between urban and rural areas” (suburban), or “village/ special area” (rural) (valid frequencies were 3541, 1411, and 14,549, respectively); (4) employment sector with responses of “non-agricultural,” “agricultural employed,” or “unemployed” (valid frequencies were 991, 1267, and 17,558, respectively); (5) education with responses of “illiterate,” “primary school,” “middle school,” “high school/vocational school”, or “college” (valid frequencies were 4496, 8484, 4318, 2088, and 430, respectively); and (6) health status with responses of “good” or “not good” (valid frequencies were 4577 and 13,701, respectively).

#### Geographic control

Since respondents come from different geographic regions (i.e., villages), and respondents within the same locale might have similarities in socio-economic, cultural backgrounds and behavioral patterns without being independent, we also included a geographic measure (‘communityID’) to reflect different geographic areas for the studied subjects. There were a total 450 communities or villages in the dataset.

### Analyses

Quantitative variables were summarized by means and standard deviations. Qualitative variables were summarized by percentages, and chi-square tests were used to compare the percentages among groups. Multiple-factor generalized linear mixed model was applied to estimate the odds ratio (OR) and the 95% confidence interval (CI) of HI coverage for the four indicators of access to care, after controlling for individual characteristics and aggregation among different rural villages and urban neighborhoods. All analyses were performed using SPSS 24.0 software.

## Results

Of the 19,816 subjects selected from the CHARLS data, 757 had missing values for the variable of insurance coverage. The subjects’ average age was 61.74 ± 10.33 (range of 18–118) years.

### Bivariate analysis of health insurance status and individual characteristics

Based on the values in Table 1, subjects’ age and gender had no significant association with reported types of HI. However, residence, occupation, education, and health status were significantly associated with insurance type (*p* < 0.001). Subjects who resided in rural area had the highest proportion of low-coverage insurance (72.3%) and no insurance (3.4%). Subjects who resided in urban area had the highest proportion of high-coverage insurance (38.6%). Subjects who resided in suburban area had the highest proportion of moderate-coverage insurance (22.7%). Adults employed within the agricultural sector had higher percentages of low-coverage insurance when compared to adults employed within the non-agricultural sector (69.4% vs. 60.9%). Adults employed within the non-agricultural sector had higher percentages of moderate-coverage insurance when compared to adults employed within the agricultural sector (21.6% vs. 13.8%). There was a gradient association between education and insurance type. Those with college or above education had the highest high-coverage rate (46.0%), followed by those with high/vocational school education (27.4%), middle school education (18.9%), primary school education (12.1%), and no education (9.8%). Adults with a middle school, high school, or college education reported lower rates of being uninsured. Those with ‘good health’ were more likely than those with ‘not-good health’ (referred to as ‘poor health’), to have high-coverage (17.9% vs. 14.3%) and moderate-coverage (18.3% vs. 16.3%) insurance. Those with ‘poor health’ were more likely than those with ‘good health’ to have low-coverage (66.1% vs. 61.1%) and no (3.2% vs. 2.7%) insurance.

**Table 1:** Health insurance status and individual characteristics

***Insurance Status:*** ***Personal Characteristics:***	Total	High-coverage Insurance	Moderate-coverage Insurance	Low-coverage Insurance	No insurance	Chi-square *p*-value
Age						4.736, 0.192
< 64	11,530	1721 (14.9%)	1929 (16.7%)	7520 (65.2%)	360 (3.1%)	
≥ 65	7216	1160 (16.1%)	1206 (16.7%)	4623 (64.1%)	227 (3.1%)	
Gender						1.359, 0.715
Male	8976	1372 (15.3%)	1527 (17.0%)	5791 (64.5%)	286 (3.2%)	
Female	10,083	1550 (15.4%)	1656 (16.4%)	6564 (65.1%)	313 (3.1%)	
Residence						2159.896, < 0.001
Urban	3367	1298 (38.6%)	652 (19.4%)	1344 (39.9%)	73 (2.2%)	
Suburban	1354	302 (22.3%)	308 (22.7%)	704 (52.0%)	40 (3.0%)	
Rural	14,040	1256 (8.9%)	2157 (15.4%)	10,149 (72.3%)	478 (3.4%)	
Employment						39.017, < 0.001
Non-agricultural	954	122 (12.8%)	206 (21.6%)	581 (60.9%)	45 (4.7%)	
Agricultural	1236	183 (14.8%)	170 (13.8%)	858 (69.4%)	25 (2.0%)	
Unemployed	16,869					
Education						778.798, < 0.001
Illiterate	4357	426 (9.8%)	671 (15.4%)	3115 (71.5%)	145 (3.3%)	
Primary school	8169	986 (12.1%)	1383 (16.9%)	5500 (67.3%)	300 (3.7%)	
Middle school	4160	786 (18.9%)	714 (17.2%)	2552 (61.3%)	108 (2.6%)	
High school/Vocational school	1973	540 (27.4%)	353 (17.9%)	1039 (52.7%)	41 (2.1%)	
College	400	184 (46.0%)	62 (15.5%)	149 (37.2%)	5 (1.2%)	
Health status						51.223, < 0.001
Good	4406	788 (17.9%)	805 (18.3%)	2692 (61.1%)	121 (2.7%)	
Not good	13,217	1888 (14.3%)	2158 (16.3%)	8742 (66.1%)	429 (3.2%)	

### Bivariate analysis of health insurance status and access to care

Table 2 shows that the status of physical examination, inpatient care, and satisfaction were significantly different among the four types of HI. Subjects without insurance, when compared with their insured counterparts, had a higher proportion of never having had a physical examination, a higher proportion of not having received inpatient care (91.5% > 79.6, 83.5, 83.4%), and a higher proportion of unsatisfactory experiences with medical care (20.0% > 17.3, 15.6, 16.1%). Among those with HI, there was a gradient association between extent of coverage and access to care. Individuals with high-coverage were more likely to have physical exam and medical office visit than those with moderate-coverage (69.7% vs. 51.1, 17.0% vs. 16.2%, respectively). Individuals with moderate-coverage were more likely to have physical exam and medical office visit than those with low-coverage (51.1% vs. 41.9, 16.2% vs. 16.1%, respectively).

**Table 2:** Health insurance status and access to care

***Insurance Status:*** ***Access to care:***	High-coverage Insurance	Moderate-coverage Insurance	Low-coverage Insurance	No insurance	Chi-square *p*-value
Physical exam					824.565, < 0.001
Have (*n* = 8856)	1979 (69.7%)	1604 (51.1%)	5110 (41.9%)	163 (27.6%)	
Have never (*n* = 9916)	862 (30.3%)	1532 (48.9%)	7095 (58.1%)	427 (72.4%)	
Medical office visit					6.539, 0.088
Yes (*n* = 3080)	498 (17.0%)	515 (16.2%)	1990 (16.1%)	77 (12.9%)	
No (*n* = 15,979)	2424 (83.0%)	2668 (83.8%)	10,365 (83.9%)	522 (87.1%)	
Inpatient care					55.795, 0.001
Yes (*n* = 3225)	595 (20.4%)	525 (16.5%)	2054 (16.6%)	51 (8.5%)	
No (*n* = 15,826)	2326 (79.6%)	2657 (83.5%)	10,296 (83.4%)	547 (91.5%)	
Satisfaction with quality, cost, and convenience					8.925, 0.030
Satisfied (*n* = 15,321)	2324 (82.7%)	2593 (84.4%)	9961 (83.9%)	443 (80.0%)	
Dissatisfied (*n* = 2994)	486 (17.3%)	480 (15.6%)	1917 (16.1%)	111 (20.0%)	

### Generalized linear mixed model of health insurance status and access to care

Table 3 presents multiple-factor generalized linear mixed model results of health insurance status and access to care. After controlling for the individual characteristics in this model, we found that individuals with HI were more likely to have completed checkup (ORs: 5.464 [4.370–6.831], 2.462 [1.983–3.057], 1.819 [1.485–2.229]), attended physician office visit (ORs: 1.496 [1.125–1.990], 1.398 [1.056–1.852],1.344 [1.040–1.752]), and received inpatient care (ORs: 2.572 [1.862–3.552], 2.065 [1.498–2.846], 2.068 [1.520–2.812]). But we only found that individuals with low-coverage insurance were more likely to have experienced satisfaction with the quality of care (OR: 1.263 [1.002–1.592]) than individuals with no insurance.

**Table 3:** Multi-logistic regression model of health insurance status and access to care

	Physical examination (have vs. have never) OR (95% CI)	Office visit (yes vs. no) OR (95% CI)	Inpatient care (yes vs. no) OR (95% CI)	Satisfied with quality (satisfied vs. dissatisfied) OR (95% CI)
***Insurance Status:***				
High-coverage vs. uninsured	5.464 (4.370–6.831)*	1.496 (1.125–1.990)*	2.572 (1.862–3.552)*	1.269 (0.983–1.639)
Moderate-coverage vs. uninsured	2.462 (1.983–3.057)*	1.398 (1.056–1.852)*	2.065 (1.498–2.846)*	1.237 (0.963–1.589)
Low-coverage vs. uninsured	1.819 (1.485–2.229)*	1.344 (1.030–1.752)*	2.068 (1.520–2.812)*	1.263 (1.002–1.592)*
Age				
< 64 vs. ≥65	0.996 (0.928–1.069)	1.027 (0.939–1.124)	1.053 (0.963–1.151)	1.087 (0.991–1.191)
Gender				
Male vs. female	0.969 (0.905–1.037)	0.993 (0.910–1.084)	0.975 (0.894–1.063)	1.001 (0.916–1.094)
Residence				
Urban vs. Rural	1.005 (0.894–1.130)	0.926 (0.808–1.060)	0.929 (0.814–1.062)	0.919 (0.800–1.054)
Suburban vs. Rural	0.935 (0.810–1.079)	0.989 (0.833–1.175)	1.011 (0.854–1.196)	0.971 (0.814–1.159)
Employment				
Non-agriculture vs. unemployed	1.061 (0.916–1.228)	0.679 (0.547–0.843)*	1.010 (0.838–1.218)	0.960 (0.793–1.162)
Agriculture vs. unemployed	1.029 (0.904–1.172)	1.082 (0.919–1.273)	1.004 (0.851–1.184)	1.032 (0.869–1.226)
Education				
Elementary school vs. illiterate	1.202 (0.922–1.566)	1.073 (0.780–1.477)	1.091 (0.800–1.488)	0.824 (0.597–1.137)
Middle school vs. illiterate	1.179 (1.027–1.355)	0.987 (0.827–1.178)	1.054 (0.889–1.251)	0.857 (0.717–1.025)
High school vs. illiterate	1.052 (0.942–1.175)	0.966 (0.839–1.113)	0.988 (0.861–1.135)	0.903 (0.781–1.043)
College vs. illiterate	0.991 (0.906–1.084)	1.028 (0.917–1.152)	0.967 (0.863–1.082)	0.915 (0.812–1.032)
Health status				
Good vs. not good	0.997 (0.925–1.074)	0.892 (0.809–0.984)*	0.855 (0.775–0.942)*	1.082 (0.980–1.195)

*: *P* < 0.05

The magnitude of the odds ratios also demonstrated a gradient association between extent of insurance coverage and access to care. Those with high-coverage had the highest odds ratios, followed by those with moderate-coverage and low-coverage, for checkup (5.464 vs. 2.462 vs. 1.819), office visit (1.496 vs. 1.398 vs. 1.344), and inpatient care (2.572 vs. 2.065 vs. 2.068). The association between extent of coverage and satisfaction with care was less pronounced.

## Discussion

In contrast to the HI system established within the United States, China’s social HI system is provided by and paid for by the state [27–29]. China’s multi-level social HI system includes urban employee basic medical insurance (UEBMI) for the urban employed population and retirees, urban resident basic medical insurance (URBMI) for the urban unemployed population, and the new cooperative medical scheme (NCMS) for the rural population [20]. The UEBMI is considered most generous and, along with government medical insurance for government employees and private medical insurance either purchased by work unit or individuals, cover about 80% of the cost of medical care. URBMI is considered a moderate-coverage HI and, along with urban and rural resident medical insurance developed in advanced communities, cover 50–80% of the cost of medical care. NCMS is considered low-coverage HI and, along with the new rural cooperative medical insurance (he-zuo-yi-liao), cover less than 50% of the cost of medical care.

Our study analyzed data from CHARLS 2018 survey and found that the majority of Chinese adults had some HI coverage. In 2018, 3.15% of Chinese adults were uninsured, compared to about 13% of US adults who were uninsured in the same year [11].

One important finding of our study was that HI coverage was significantly and positively associated with access to care. Specifically, we observed that Chinese adults with HI were more likely to have physical examinations, office visits, and inpatient care than those with no insurance- a finding which was similar to studies of the US [16, 17, 20, 30] and India [31], even though these latter studies examined different indicators related to access to care [16, 30, 31]. Researchers in the US [16] showed that CHC patients with insurance coverage were more likely to have access to necessary medical care (OR = 2.12), see a recommended specialist (OR = 2.73), see a mental health professional if advised to do so (OR = 1.74), receive recommended follow-up care after an abnormal pap smear (OR = 3.44), and obtain necessary prescription medications (OR = 2.10) (particularly high cholesterol medications (OR = 2.25), compared to similar CHC patients without insurance. Among privately- and publicly-insured cancer survivors, those with coverage disruptions were less likely to report all preventative service use (16.9% vs. 36.2%; 14.6% vs. 25.3%, respectively) and more likely to report problems with care affordability (55.0% vs. 17.7%; 71.1% vs. 38.4%, respectively) and cost-related medication nonadherence (39.4% vs. 10.1%; 36.5% vs. 16.3%, respectively), compared with those continuously insured [32]. The results from another US study suggested that the high uninsured rate among Mexican migrant workers in the US contributed to inadequate access to care in the country overall [20]. A further US study noted that the Medicaid expansion resulted in substantial increases in insurance coverage and was associated with significant reductions in delaying care [17]. In the study from India [33], researchers found that individuals enrolled in HI specified for the poor had 1.21 times higher odds of hospitalization incidences when compared to poor individuals without HI coverage. Researchers analyzed the piloting of a preferred provider system (PPS) for rural members of Vimo SEWA, a fixed-indemnity, community-based HI (CBHI) scheme in India [2] and showed that it successfully directed members to inpatient facilities with acceptable level of technical quality [33]. A study from P.R. China showed that compared with the insured, the respondents without medical insurance had 73% less probability of using outpatient services in the past month, 49% less probability of hospitalization in the past year,67% less probability of receiving routine physical examination in the past 2 years, and 84% less probability of receiving other treatment in the past month [24].

In this study, we also found that Chinese adults with HI were more likely to be satisfied with quality of care than those with no insurance, which was different from previous study findings from Ghana [26]. The definition of satisfaction may have varied, affecting the results of our study. For example, satisfaction was measured from the following domains in a Nigerian study [34]: accessibility, patient waiting time, patient–provider communication, patient–provider relationship, hospital bureaucracy, and hospital environment. Patients were then categorized in the binary, with those who scored 50% and above in the assessed domain were considered satisfied, while those who scored less than 50% were dissatisfied [34]. In our study, Chinese interviewees were simply asked if they were satisfied with “the quality, cost, and convenience of local medical services.” However, the result of our study was consistent with another China study which showed that community elderly chronic patients with medical insurance were more satisfied with service quality and medical expenses than patients without medical insurance (76.28% > 61.11%, 81.01% > 59.30%, respectively) [35].

Perhaps the most important finding of our study was that there was a significant gradient association between extent of insurance coverage and access to care. We found that adults with high-coverage HI had much higher odds of having regular checkup when compared to adults with moderate-coverage or low-coverage (ORs with 95% CI: 5.464 [4.370–6.831] > 2.462 [1.983–3.057] > 1.819 [1.485–2.229]). Likewise, when compared to adults with moderate-coverage or low-coverage, those with high-coverage HI had higher odds of having physician office visits (ORs with 95% CI: 1.496 [1.125–1.990], 1.398 [1.056–1.852],1.344 [1.040–1.752]) and inpatient care (ORs with 95% CI: 2.572 [1.862–3.552], 2.065 [1.498–2.846], 2.068 [1.520–2.812]). This result implies that while HI is important in general, adequate coverage is also essential to improve access to care particularly preventive care (our checkup measure), primary care (our physician office visit measure), and tertiary care (our inpatient care measure). Alternatively, this finding could also indicate the presence of a sizable number of Chinese individuals who remain under-insured and thereby experience barriers to and inaccessibility of healthcare.

There were a number of limitations with this study. The cross-sectional nature of the data makes it impossible to assess causality. Therefore, our findings demonstrated associations rather than causal relationships. We were also limited by the number of measures within CHARLS which restricted the analyses performed. Noticeably absent were quality of care and cost of care measures, both critical in assessing the impact of HI. Finally, the impact of COVID was not assessed by the dataset. Under COVID type of insurance coverage and payment could greatly affect access to healthcare. Future research should look at longitudinal effect of extent of insurance coverage on access, quality, and cost of healthcare and under circumstances like COVID.

Insurance status in China is also tied to patient satisfaction, the standard by which healthcare quality is assessed. Patients with adequate insurance and fewer incidences of catastrophic hospital costs are more satisfied. Higher satisfaction rates have also been observed in patients with UEBMI insurance status [32, 33]. The fulfillment of patients’ expectations of insurance benefits and coverage was the major predictor of patient satisfaction. However, this expectation of insurance benefits varies from rural to urban populations. In a 2016 study of 1200 respondents, rural patients were noted to have lower expectations for the insurance plan and as a result, significantly higher levels of satisfaction with care received when compared to their urban counterparts. Beyond expectations for insurance coverage, urban patients’ levels of satisfaction was also tied to convenience of hospital location, more choices of treatments, and more thorough physical check-ups [32–34].

In sum, our study is one of few recent studies that used a country’s representative sample survey to study the association between extend of HI coverage and access to needed healthcare (preventive, primary, and tertiary). We showed that not only HI mattered in enhancing access to care but that there was a significant gradient association between extent of HI coverage and access to care with higher coverage relating to better access.

## Data Availability

The datasets generated and analyzed during the current study are available in the CHARLS data repository (http://charls.pku.edu.cn/pages/about/charls/zh-cn.html).
